# Outcomes of patients with hematological malignancies who undergo unrelated donor hematopoietic stem cell transplantation with ATG-Fresenius versus ATG-Genzyme

**DOI:** 10.1007/s00277-023-05220-7

**Published:** 2023-04-25

**Authors:** Lu Wang, Peiyan Kong, Cheng Zhang, Li Gao, Lidan Zhu, Jia Liu, Shichun Gao, Ting Chen, Huanfeng Liu, Han Yao, Yuqing Liu, Yimei Feng, Lu Zhao, Yuxia Li, Lei Gao, Xi Zhang

**Affiliations:** Medical Center of Hematology, Xinqiao Hospital, Army Medical University, Chongqing, China

**Keywords:** Anti-thymocyte globulin, Allogeneic hematopoietic stem cell transplantation, Unrelated donor, Graft-versus-host disease

## Abstract

To compare the outcomes of patients with hematological malignancies who received ATG-Fresenius (ATG-F) 20 mg/kg versus those who received ATG-Genzyme (ATG-G) 10 mg/kg in an unrelated donor hematopoietic stem cell transplantation (HSCT) procedure, a total of 186 patients who underwent their first allogeneic HSCT with an unrelated donor were retrospectively analyzed. One hundred and seven patients received ATG-F, and seventy-nine patients received ATG-G. Multivariate analysis showed that the type of ATG preparation had no effect on neutrophil engraftment (*P* = 0.61), cumulative incidence of relapse (*P* = 0.092), nonrelapse mortality (*P* = 0.44), grade II-IV acute graft-versus-host disease (GVHD) (*P* = 0.47), chronic GVHD (*P* = 0.29), overall survival (*P* = 0.795), recurrence-free survival (*P* = 0.945) or GVHD-free relapse-free survival (*P* = 0.082). ATG-G was associated with a lower risk of extensive chronic GVHD and a higher risk of cytomegaloviremia (*P* = 0.01 and HR = 0.41, *P* < 0.001 and HR = 4.244, respectively). The results of this study suggest that the preparation of rabbit ATG used for unrelated HSCT should be selected based on the incidence of extensive chronic GVHD of each center, and the posttransplant management strategy should be adjusted according to the ATG preparation.

## Introduction

Allogeneic hematopoietic stem cell transplantation is an important method to cure hematological malignancies, and unrelated donors are important alternatives for patients without HLA identical sibling donors. Anti-thymocyte globulin (ATG) can decrease the incidence of graft-versus-host disease (GVHD) in HLA-matched and HLA-mismatched unrelated transplantation and improves the survival of mismatched unrelated transplantation [1–10]. Two commercial preparations of rabbit ATG, ATG-Fresenius (ATG-F, currently sold as ATG-Grafalon) and ATG-Genzyme (ATG-G), are widely used in hematopoietic stem cell transplantation. ATG-G is manufactured by rabbit immunization against human thymocytes, whereas ATG-F is produced by immunizing rabbits with the Jurkat human T-lymphoblastic cell line [11]. The different manufacturing methods result in discrepancies in antibody specificities and immunomodulatory effects independent of their ability to deplete T cells [11–13]. Comparisons of their protective role in unrelated transplantation have been conducted in several studies, but the conclusions are varied [14–18]. In two of these studies, the doses of both products were not fixed [14, 15], so the findings are difficult to interpret. The remaining studies compared ATG-F and ATG-G at fixed doses; however, the case number was too small, and multivariate analyses were not conducted [16–18]. Therefore, studies are still needed to compare the efficacy of ATG-F and ATG-G at fixed doses. In this study, we retrospectively analyzed 186 unrelated donor transplantation patients from a single center who received ATG-F 20 mg/kg or ATG-G 10 mg/kg in their transplantation procedure and compared the outcomes of patients who received different ATGs.

## Patients and methods

### Patients

From August 2007 to May 2021, a total of 186 patients diagnosed with malignant hematological diseases without matched sibling donors underwent their first allo-SCT procedure at Xinqiao Hospital, Army Medical University, with an HLA-matched (10/10) or mismatched (9/10 or 8/10) unrelated donor following ATG-containing conditions. All donors were HLA fully matched (10/10) or mismatched at one or two loci (9/10 or 8/10) (HLA-A, B, C, DRB1, DQB1) by high-resolution HLA typing. Patients who previously underwent allogenic transplantation were excluded. This study was approved by the Ethics Committee of Xinqiao Hospital, Army Medical University, and written informed consents were obtained from all patients before transplantation.

### Transplantation procedure

The conditioning regimen included TBI/CY, BU/CY, CCNU/MeCCNU + Ara-c + BU + CY (usually used in haploidentical transplantation in China [19]), FB3 or other regimens. For patients who received TBI/CY, 8–9.5 Gy total body irradiation was delivered and fractioned by two days, and a total dose of 120 mg/kg cyclophosphamide was administered. For patients who received BU/CY, a total dose of 12.8 mg/kg intravenous busulfan and 120 mg/kg cyclophosphamide was administered. For patients who received CCNU/MECCNU + Ara-c + BU + CY, 200 mg/m^2^ lomustine or semustine, a total dose of 8 g/m^2^ cytarabine, 9.6 mg/kg intravenous busulfan and 3.6 g/m^2^ cyclophosphamide was administered. For patients who received FB3, a total dose of 150 mg/m^2^ fludarabine and 390 mg/m^2^ busulfan was administered. Every patient received a total dose of 10 mg/kg ATG-G or 20 mg/kg ATG-F as part of their conditioning regimen. All patients received unmanipulated granulocyte colony-stimulating factor–mobilized peripheral blood mononuclear cells on day 0 and received cyclosporine/tacrolimus, mycophenolate mofetil and low-dose methotrexate for GVHD prophylaxis. The dose of cyclosporine was adjusted to maintain a trough serum concentration of 150–300 µg/ml and the dose of tacrolimus was adjusted to achieve a trough serum concentration of 5–15 ng/ml. Cyclosporine and tacrolimus were tapered beginning at days + 100 depending on GVHD status. Mycophenolate mofetil was taken orally from day 0 at a dose of 600 mg/m^2^ per day in divided doses and was tapered to discontinuation between days + 30 and + 60. MTX was administered intravenously at a dose of 15 mg/m^2^ on days + 1 and 10 mg/m^2^ on days + 3, + 6 and + 11. Cytomegalovirus (CMV) DNA in blood samples was monitored weekly by real-time PCR. Once the CMV copies were more than 400/ml in two independent tests, ganciclovir or foscarnet combined with γ-globulin was given. Some patients received posttransplant maintenance therapy to prevent relapse, including tyrosine kinase receptor inhibitors for patients diagnosed with CML, Philadelphia chromosome-positive ALL and AML with KIT mutation, demethylating agents for patients diagnosed with AML without target drugs available, and chidamide for patients diagnosed with T-ALL.

### Definition of disease stage

Disease stage was defined according to our and others’ published literature [5, 20, 21]. Early-stage disease was defined as CML in the first chronic phase, de novo acute leukemia in CR1, MDS-RA, MDS-RARS, CLL and lymphoma with chemotherapy-sensitive disease or the most recent relapse-free interval greater than 6 months. Late-stage disease was defined as CML in the accelerated phase or in the second chronic phase, secondary/therapy-related acute leukemia in CR1, acute leukemia in the second or third remission, MDS-EB, lymphoma with disease that was not regarded as chemotherapy-sensitive or the most recent relapse interval was 6 months or less. Active disease was defined as CML in the blast phase, acute leukemia without remission, and lymphoma with over 20% tumor cells in the bone marrow.

### Statistics

Patient characteristics are expressed as the median and range for continuous variables, and the difference between groups was tested by the Mann–Whitney method. Categorical variables are expressed as frequencies. The differences between groups were tested by the chi square or Fisher’s exact test, and multivariate analysis was conducted by a logistical regression model.

Overall survival (OS) was measured from transplantation to death from any cause. Recurrence-free survival (RFS) was defined as survival without disease recurrence. GVHD-free relapse-free survival (GRFS) was defined as survival without grade III-IV acute GVHD (aGVHD) or chronic GVHD (cGVHD) requiring systematic treatment or disease recurrence. OS, RFS and GRFS were estimated by the Kaplan–Meier method. Univariate comparisons were performed using the log-rank test, and the Cox proportional hazards regression model was used for multivariate analysis.

Neutrophil engraftment was defined as an absolute neutrophil count of at least 500/µl for 3 consecutive days after transplantation. Neutrophil engraftment at days +28, cumulative incidence of relapse (CIR), nonrelapse mortality (NRM), aGVHD and cGVHD were estimated by a competing risk model. Death was regarded as a competing event for neutrophil engraftment, aGVHD and cGVHD. In addition, DLI and secondary transplantation were also considered competing events for aGVHD and cGVHD. NRM and relapse were competing events for each other. Univariate significance was estimated by Gray’s K-sample test, and multivariate analysis was conducted by competing risk regression.

All factors with a *P* value < 0.1 by univariate analysis were included in the multivariate analysis. In addition, ATG preparation and HLA match were entered into the multivariate analysis regardless of the P value in the univariate analysis. Mann–Whitney, chi square test, Fisher’s exact test, logistical regression model, Kaplan–Meier and Cox regression models were performed with SPSS 23.0. Cumulative incidences were computed with R functions from the package cmprsk (R version × 64 4.1.2, package cmprsk version 2.2–11).

## Results

### Patient characteristics

A total of 186 patients were included in this study. One hundred and seven patients received ATG-F and seventy-nine patients received ATG-G. There were no significant differences between the patients who received ATG-F and the patients who received ATG-G with respect to patient sex, age, disease stage, donor-recipient sex, conditioning regimen, HLA match, infused mononuclear cell number, infused CD34 + cell number or GVHD prophylaxis (Table 1). The patient diagnosis was significantly different between the ATG-F group and the ATG-G group, with acute leukemia with ambiguous lineage (ALAL), acute lymphoblastic leukemia (ALL), acute myeloid leukemia (AML), chronic lymphocytic leukemia (CLL), chronic myelogenous leukemia (CML), marrow dysplastic syndrome (MDS) and non-Hodgkin’s lymphoma (NHL) proportions of 3.7% vs. 5.1%, 27.1% vs. 20.3%, 39.3% vs. 54.4%, 0.9% vs. 0%, 21.5% vs. 7.6%, 6.5% vs. 7.6%, and 0.9% vs. 5.1%, respectively (*P* = 0.032).

**Table 1:** Patient characteristics

		ATG-F		ATG-G		*P* value
		No	%	No	%	
Sex, n (%)	Male	63	58.9%	50	63.3%	0.542
	Female	44	41.1%	29	36.7%	
Age	Median (range)	25 (3–59)		30 (3–65)		0.103
Diagnosis	ALAL	4	3.7%	4	5.1%	0.032
	ALL	29	27.1%	16	20.3%	
	AML	42	39.3%	43	54.4%	
	CLL	1	0.9%	0	0.0%	
	CML	23	21.5%	6	7.6%	
	MDS	7	6.5%	6	7.6%	
	NHL	1	0.9%	4	5.1%	
Disease stage	Early stage	80	74.8%	63	79.7%	0.762
	Late stage	20	18.7%	12	15.2%	
	Active disease	7	6.5%	4	5.1%	
Donor-recipient sex	Male to male	47	43.9%	42	53.2%	0.356
	Male to female	34	31.8%	25	31.6%	
	Female to male	16	15.0%	9	11.4%	
	Female to female	10	9.3%	3	3.8%	
Conditioning regimen	TBI/CY	10	9.3%	3	3.8%	0.518
	BU/CY	60	56.1%	49	62.0%	
	Haplo regimen	30	28.0%	21	26.6%	
	FB3	6	5.6%	6	7.6%	
	Other	1	0.9%	0	0.0%	
HLA match	Matched	69	64.5%	45	57.0%	0.298
	Mismatched	38	35.5%	34	43.0%	
MNC	Median (range)	9.5 (3.2–33.4)		8.73 (4.0–17.2)		0.131
	NA	5		4		
CD34 + cells	Median (range)	6.3 (1.1–31.6)		5.55 (1.2–20.6)		0.057
	NA	8		3		
GVHD prophylaxis	CSA + MMF + MTX	96	89.7%	67	84.8%	0.315
	FK506 + MMF + MTX	11	10.3%	12	15.2%	

The haplo regimen indicates CCNU/MeCCNU + Ara-c + BU + CY

### Engraftment

**Fig. 1:**
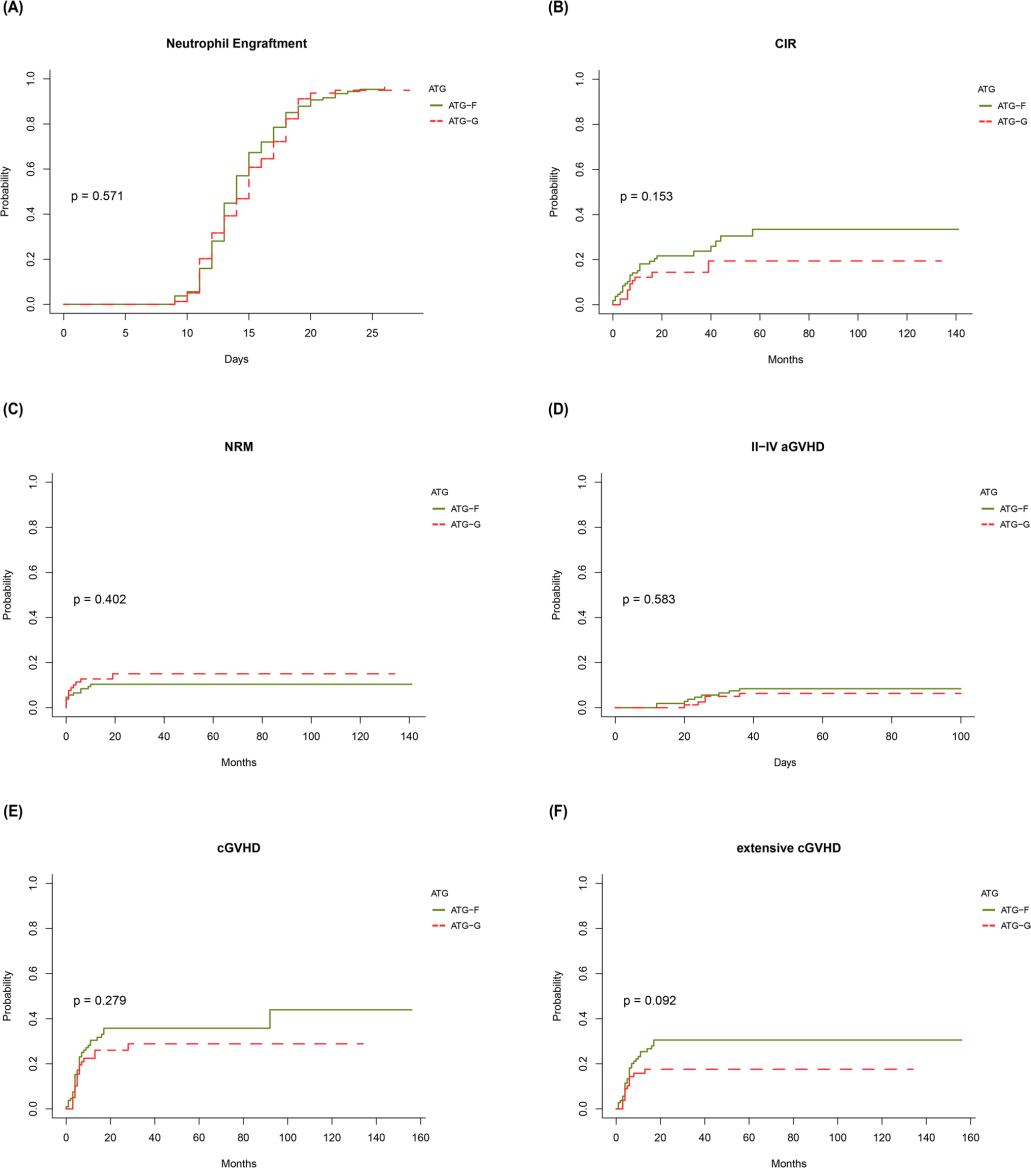
**A** Neutrophil engraftment at days +28, **B** cumulative incidence of relapse, **C** nonrelapse mortality, **D** cumulative incidence of grade II-IV acute GVHD, **E** cumulative incidence of chronic GVHD and **F** cumulative incidence of extensive chronic GVHD for patients receiving ATG-Fresenius and patients receiving ATG-Genzyme

The cumulative incidence of neutrophil engraftment at days +28 in the ATG-F group and the ATG-G group was similar (96.3% vs. 94.9%, *P* = 0.571, Fig. 1A). Multivariate analysis showed that the type of ATG preparation had no impact on neutrophil engraftment (*P* = 0.61, Table 3). Receiving CCNU/MECCNU + Ara-c + BU + CY as a conditioning regimen was an independent risk factor for neutrophil engraftment (HR = 0.607 and *P* = 0.005, Table 3).

### Relapse and nonrelapse mortality

CIR in the ATG-F group and the ATG-G group was not significantly different (33.5% vs. 19.4%, *P* = 0.153, Fig. 1B). Factors affecting the CIR included pretransplant disease stage, GVHD prophylaxis, cGVHD and maintenance therapy (*P* = 0.043, *P* = 0.0495, *P* = 0.008, *P* = 0.049, respectively, Table 2). Multivariate analysis showed that the type of ATG preparation had no effect on CIR (*P* = 0.092, Table 3). Pretransplant active disease and the use of FK506 + MMF + MTX as GVHD prophylaxis were independent risk factors for relapse (HR = 3.371 and *P* = 0.028 and HR = 2.93 and *P* = 0.024, respectively, Table 3), while extensive cGVHD was a preventative factor for relapse (HR = 0.23, *P* = 0.017, Table 3).

**Table 2:** Univariate analysis for neutrophil engraftment, CIR, NRM, II-IV aGVHD, cGVHD, extensive cGVHD and cytomegalovirusemia

		Neutrophil engraftment		CIR		NRM		II-IV aGVHD		cGVHD		Extensive cGVHD		Cytomegalovirusemia	
		%	*P* value	%	*P* value	%	*P* value	%	*P* value	%	*P* value	%	*P* value	%	*P* value
ATG	ATG-F	96.3	0.571	33.5	0.153	10.4	0.402	8.4	0.583	43.9	0.279	30.5	0.092	29.9	< 0.001
	ATG-G	94.9		19.4		15		6.3		28.8		17.6		64.6	
Sex	Male	94.7	0.235	28.7	0.382	15.9	0.088	8	0.779	46.3	0.933	22.1	0.3	41.6	0.301
	Female	97.3		27.5		6.9		6.8		33.9		30.4		49.3	
Age group	< = 20	93.9	0.918	20.7	0.488	10.7	0.911	4.5	0.408	40.1	0.66	24.2	0.792	36.4	0.083
	> 20, < = 40	98.7		34.1		13		8		37.1		28.3		44	
	> 40	93.3		32.7		13.4		11.1		27.4		22.7		57.8	
Disease stage	Early stage	95.8	0.291	26.1	0.043	11.4	0.25	9.8	0.104	39.2	0.201	26	0.188	45.5	0.892
	Late stage	93.8		31.6		10.6		0		41.1		30.9		43.8	
	Active disease	100		54.5		27.3		0		18.9		0		36.4	
Condition	TBI/CY	100	0.056	49.2	0.135	7.7	0.825	7.7	0.328	46.2	0.494	23.1	0.89	23.1	0.09
	BU/CY	97.2		21.8		11.6		5.5		45.4		27.9		52.3	
	Haplo regimen	94.1		31.4		15.7		13.7		26.8		22.5		37.3	
	FB3	83.3		42.3		8.3		0		23.6		23.6		33.3	
HLA	Matched	94.7	0.419	26.1	0.531	12	0.8	4.4	0.041	45.3	0.294	26.7	0.635	45.6	0.732
	Mismatched	97.2		32.6		12.6		12.5		27.7		23.4		43.1	
GVHD prophylaxis	CSA + MMF + MTX	95.1	0.479	25.8	0.0495	12.1	0.88	7.4	0.805	39.9	0.733	26.7	0.245	43.6	0.437
	FK506 + MMF + MTX	100		50.5		13.3		8.7		31.1		15.6		52.2	
aGVHD	No or 0-I	–	–	27.9	0.96	10.5	0.007			38.6	0.422	24	0.184	42	0.058
	II	–	–	25		12.5				50		50		62.5	
	III-IV	–	–	31.1		48.9				52.8		40		77.8	
cGVHD	No	–	–	34.8	0.008	14.1	0.213			–	–	–	–	41.9	0.132
	Limited	–	–	16.8		16.1				–	–	–	–	30.8	
	Extensive	–	–	12.2		6				–	–	–	–	56.8	
Maintenance therapy	No	–	–	31.5	0.049	14	0.104			39.2	0.588	25.3	0.607	40	0.005
	Yes	–	–	7.9		3.3				32.8		26.3		67.7	
Cytomegalovirusemia	No	–	–	33.8	0.225	15.1	0.201			37.3	0.147	18.8	0.03		
	Yes	–	–	22.2		8.6				39.4		33.9			

The haplo regimen indicates CCNU/MeCCNU + Ara-c + BU + CY

**Table 3:** Multivariate analysis for neutrophil engraftment, CIR, NRM, II-IV aGVHD,cGVHD, extensive cGVHD and cytomegalovirusemia

		Variable	*P* value	HR (95% CI)
Neutrophil engraftment	ATG	ATG-G vs. ATG-F	0.61	0.933 (0.714–1.219)
	HLA	Mismatched vs. matched	0.55	1.105 (0.799–1.527)
	Conditioning regimen	TBI/CY vs. BU/CY	0.62	0.885 (0.545–1.438)
		Haplo regimen vs. BU/CY	0.005	0.607 (0.427–0.862)
		FB3 vs. BU/CY	0.12	0.56 (0.272–1.152)
CIR	ATG	ATG-G vs. ATG-F	0.092	0.558 (0.283–1.1)
	HLA	Mismatched vs. matched	0.57	0.808 (0.388–1.68)
	Disease stage	Late stage vs. early stage	0.37	1.517 (0.61–3.777)
		Active disease vs. early stage	0.028	3.371 (1.144–9.933)
	GVHD prophylaxis	FK506 + MMF + MTX vs. CSA + MMF + MTX	0.024	2.93 (1.151–7.455)
	cGVHD	Limited cGVHD vs. No cGVHD	0.088	0.179 (0.025–1.291)
		Extensive cGVHD vs. No cGVHD	0.017	0.23 (0.069–0.773)
	Maintenance therapy	Received vs. not received	0.094	0.334 (0.093–1.205)
NRM	ATG	ATG-G vs. ATG-F	0.44	1.394 (0.603–3.22)
	HLA	Mismatched vs. matched	0.88	0.934 (0.398–2.19)
	Recipient sex	Female vs. male	0.1	0.414 (0.143–1.2)
	aGVHD	Grade II aGVHD vs. no or grade 0-I	0.94	1.08 (0.13–8.96)
		Grade III-IV aGVHD vs. no or grade 0-I	< 0.001	5.602 (2.268–13.83)
II-IV aGVHD	ATG	ATG-G vs. ATG-F	0.47	0.675 (0.232–1.97)
	HLA	Mismatched vs. matched	0.041	3.069 (1.046–9.01)
cGVHD	ATG	ATG-G vs. ATG-F	0.29	0.751 (0.441–1.28)
	HLA	Mismatched vs. matched	0.32	0.757 (0.436–1.31)
Extensive cGVHD	ATG	ATG-G vs. ATG-F	0.01	0.41 (0.209–0.807)
	HLA	Mismatched vs. matched	0.9	0.96 (0.523–1.763)
	Cytomegaloviremia	Ocurred vs. not ocurred	0.003	2.58 (1.376–4.82)
Cytomegaloviremia	ATG	ATG-G vs. ATG-F	< 0.001	4.244(2.145–8.395)
	HLA	Mismatched vs. matched	0.917	1.043(0.472–2.303)
	Age group	> 20, < = 40 vs. < = 20	0.864	1.07(0.491–2.336)
		> 40 vs. < = 20	0.125	2.011(0.824–4.911)
	Conditioning regimen	TBI/CY vs. BU/CY	0.241	0.409(0.092–1.822)
		Haplo regimen vs. BU/CY	0.07	0.434(0.176–1.071)
		FB3 vs. BU/CY	0.106	0.306(0.073–1.288)
	aGVHD	Grade II aGVHD vs. no or grade 0-I	0.137	3.409(0.678–17.152)
		Grade III-IV aGVHD vs. no or grade 0-I	0.034	6.695(1.153–38.892)
	Maintenance therapy	Received vs. not received	0.053	2.425(0.987–5.954)

The haplo regimen indicates CCNU/MeCCNU + Ara-c + BU + CY

NRM in the ATG-F group and the ATG-G group was not significantly different (10.4% vs. 15.0%, *P* = 0.402, Fig. 1C), but it was significantly affected by acute GVHD (*P* = 0.007, Table 2). Grade III-IV aGVHD was the only risk factor for NRM in the multivariate analysis (HR = 5.602, *P* < 0.001, Table 3).

### GVHD

Univariate analysis showed that the type of ATG preparation had no effect on the cumulative incidence of grade II-IV aGVHD (8.4% vs. 6.3%, *P* = 0.583, Fig. 1D). HLA mismatch was the only factor affecting the incidence of grade II-IV aGVHD (*P* = 0.041, Table 2). Multivariate analysis showed that the type of ATG preparation had no effect on grade II-IV aGVHD (*P* = 0.47, Table 3). HLA mismatch was a risk factor for grade II-IV aGVHD (HR = 3.069, *P* = 0.041, Table 3).

Univariate analysis showed no impact of the type of ATG preparation on the cumulative incidence of cGVHD (43.9% vs. 28.8%, *P* = 0.279, Fig. 1E). Multivariate analysis showed that neither of the ATG preparations was a risk factor for cGVHD (*P* = 0.29, Table 3).

There was a trend toward a higher incidence of extensive cGVHD in patients receiving ATG-F (30.5% vs. 17.6%, *P* = 0.092, Fig. 1F), and a higher incidence of extensive cGVHD in patients with cytomegaloviremia (*P* = 0.03, Table 2). Multivariate analysis showed that ATG-G was a favorable factor and cytomegaloviremia was a risk factor for extensive cGVHD (HR = 0.41 and *P* = 0.01, and HR = 2.58 and *P* = 0.003, respectively, Table 3).

### Cytomegaloviremia

The incidence of cytomegaloviremia was significantly higher in the ATG-G group (64.6% vs. 29.9%, *P* < 0.001) and in patients receiving posttransplant maintenance therapy (*P* = 0.005, Table 2). Multivariate analysis showed that ATG-G and grade III-IV aGVHD were independent risk factors for cytomegaloviremia (HR = 4.244 and *P* < 0.001, and HR = 6.695 and *P* = 0.034, respectively, Table 3).

### Survival

**Fig. 2:**
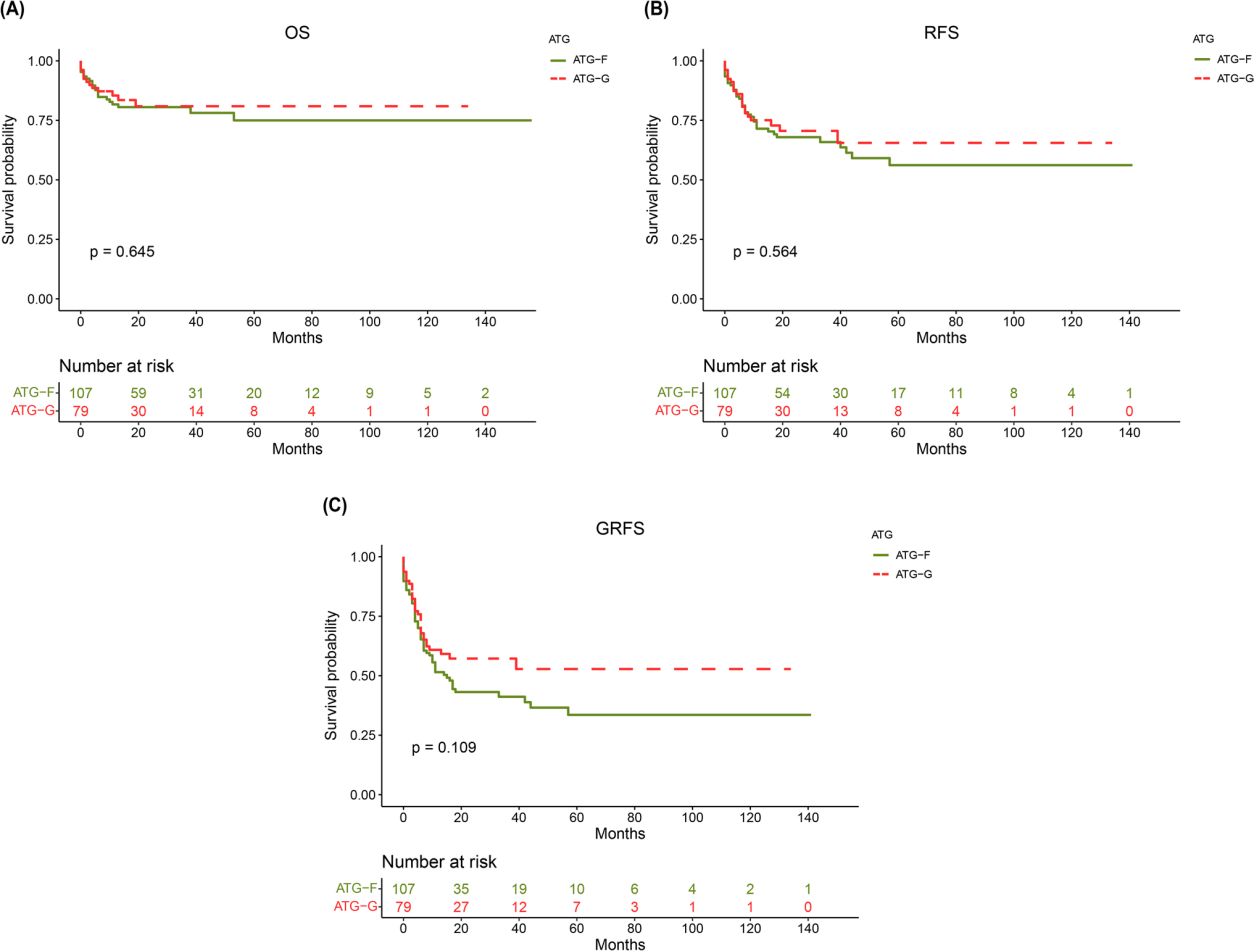
**A** Overall survival, **B** recurrence-free survival and (**C**) GVHD-free relapse-free survival for patients receiving ATG-Fresenius and patients receiving ATG-Genzyme

There was no significant difference in OS between patients receiving ATG-F and patients receiving ATG (75% vs. 80.9%, *P* = 0.645, Fig. 2A). Factors affecting OS included pretransplant disease stage, aGVHD and cGVHD (*P* = 0.001, *P* = 0.038, and *P* = 0.036, respectively, Table 4). Multivariate analysis showed that the type of ATG preparation had no impact on OS (*P* = 0.795, Table 4). Pretransplant active disease and grade III-IV aGVHD were risk factors for OS (HR = 3.462 and *P* = 0.01, and HR = 4.548 and *P* = 0.016, respectively), and extensive cGVHD was a favorable factor for OS (HR = 0.279, *P* = 0.042).

**Table 4:** Univariate and multivariate analysis for OS, RFS and GRFS

		OS				RFS				GRFS			
		%	Uni-P value	Multi-P value	HR (95% CI)	%	Uni-P value	Multi-P value	HR (95% CI)	%	Uni-P value	Multi-P value	HR (95% CI)
ATG	ATG-F	75	0.645	1	Reference	56.2	0.564	1	Reference	33.5	0.109	1	Reference
	ATG-G	80.9		0.795	1.105(0.521–2.342)	65.5		0.945	0.98(0.553–1.738)	52.8		0.082	0.686(0.448–1.048)
HLA	Matched	78.7	0.766	1	Reference	61.9	0.464	1	Reference	46.6	0.105	1	Reference
	Mismatched	73.9		0.755	0.891(0.431–1.841)	54.8		0.669	0.872(0.466–1.632)	30.7		0.081	1.437(0.957–2.158)
Sex	Male	76.1	0.118			55.4	0.052	1	Reference	36.5	0.087	1	Reference
	Female	80				65.6		0.1	0.61(0.339–1.099)	47.4		0.104	0.698(0.453–1.076)
Age group	< = 20	82.3	0.494			68.6	0.405			47.4	0.598		
	> 20, < = 40	76.5				52.9				31.6			
	> 40	68.1				53.9				40.4			
Disease stage	Early stage	80.9	0.001	1	Reference	62.5	0.001	1	Reference	44.2	0.098	1	Reference
	Late stage	79.5		0.328	1.585(0.63–3.989)	57.8		0.391	1.367(0.669–2,794)	28.5		0.38	1.263(0.75–2.126)
	Active disease	25.3		0.01	3.462(1.346–8.904)	18.2		0.025	2.607(1.129–6.019)	18.2		0.067	2.003(0.954–4.209)
Condition	TBI/CY	76.9	0.594			43,1	0.296			38.5	0.602		
	BU/CY	81.3				66.6				46.2			
	Haplo regimen	67.3				52.9				29.2			
	FB3	75.8				49.4				25.9			
GVHD prophylaxis	CSA + MMF + MTX	79.8	0.316			62.1	0.073	1	Reference	43	0.35		
	FK506 + MMF + MTX	37.8				36.2		0.099	1.924(0.885–4.18)	20			
aGVHD	No or 0-I	78.6	0.038	1	Reference	61.6	0.028	1	Reference	-	-		
	II	72.9		0.22	2.604(0.564–12.016)	62.5		0.386	1.736(0.499–6.041)	-	-		
	III-IV	46.7		0.016	4.548(1.329–15.56)	20		0.009	3.772(1.39–10.231)	-	-		
cGVHD	No	71.2	0.036	1	Reference	51.1	0.001	1	Reference	-	-		
	Limited	83.9		0.231	0.403(0.091–1.781)	67.1		0.042	0.287(0.086–0.954)	-	-		
	Extensive	91.4		0.042	0.279(0.082–0.952)	81.8		0.004	0.251(0.097–0.647)	-	-		
Maintenance therapy	No	75.2	0.056	1	Reference	54.5	0.007	1	Reference	37.8	0.193		
	Yes	82.9		0.182	0.369(0.086–1.594)	88.8		0.046	0.296(0.09–0.978)	59.9			
Cytomegalovirusemia	No	72.2	0.095	1	Reference	51.1	0.055	1	Reference	37.8	0.783		
	Yes	82.9		0.137	0.515(0.215–1.235)	69.2		0.15	0.619(0.322–1.19)	44.4			

The uni-P value indicates *P* value in the univariate analysis, the multi-P value indicates *P* value in the multivariate analysis, the haplo regimen indicates CCNU/MeCCNU + Ara-c + BU + CY

RFS did not significantly differ between patients receiving ATG-F and patients receiving ATG (56.2% vs. 65.5%, *P* = 0.564, Fig. 2B). Pretransplant disease stage, aGVHD, cGVHD and maintenance therapy were factors affecting RFS (*P* = 0.001, *P* = 0.028, *P* = 0.001, and *P* = 0.007, respectively, Table 4). Multivariate analysis showed that the type of ATG preparation did not affect RFS (*P* = 0.945, Table 4). Factors affecting RFS included pretransplant active disease (HR = 2.607, *P* = 0.025), grade III-IV aGVHD (HR = 3.772, *P* = 0.009), limited cGVHD (HR = 0.287, *P* = 0.042), extensive cGVHD (HR = 0.251, *P* = 0.004) and maintenance therapy (HR = 0.296, *P* = 0.046).

There was no significant difference in GRFS between patients receiving ATG-F and patients receiving ATG (33.5% vs. 52.8%, *P* = 0.109, Fig. 2C). Multivariate analysis showed that the type of ATG preparation was not an independent influential factor for GRFS (*P* = 0.082, Table 4).

## Discussion

In this article, we retrospectively analyzed 186 patients with hematological malignancies who underwent unrelated donor transplantation and compared the outcomes of 107 patients who received ATG-F 20 mg/kg in their transplant procedure with those of 79 patients who received ATG-G 10 mg/kg. There was no significant difference in the rates of engraftment, relapse, or NRM, the cumulative incidence of grade II-IV aGVHD or cGVHD, OS, RFS or GRFS between patients receiving ATG-F and patients receiving ATG-G. However, compared with ATG-F, ATG-G was associated with a lower risk of extensive cGVHD and a higher incidence of CMV reactivation. The more potent immunosuppressive effect of ATG-G at a dose of 10 mg/kg compared to ATG-F at a dose of 20 mg/kg may account for this finding. Two studies demonstrated that ATG-G 10 mg/kg was correlated with delayed T-cell reconstitution in comparison with ATG-F 25 mg/kg and 45–60 mg/kg [22, 23]. The broad antibody spectrum of ATG-G may also be related to its association with less extensive cGVHD and a higher rate of cytomegaloviremia. ATG-G is a polyclonal antibody that also targets molecules on B cells, such as CD19 and CD20 [24].

It is well known that ATG is associated with CMV reactivation [25], which can lead to serious complications after transplantation. Letermovir can reduce the morbidity and mortality associated with CMV reactivation, but it is too expensive for many patients in developing areas to afford. The differences in cytomegaloviremia incidence between patients receiving different ATG products suggest that the prevention of posttransplant CMV reactivation could be adjusted according to the ATG product.

The difference in the risk of extensive cGVHD between patients who received ATG-F and those who received ATG-G suggest that the selection of ATG preparation should be based on the incidence of extensive cGVHD of each center. Adjustment of the posttransplant management strategy based on ATG preparation, for example, delaying the discontinuation of calcineurin inhibitors for patients who receive ATG-Fresenius, is recommended.

In previous studies comparing ATG-F and ATG-G in unrelated transplantation at fixed doses, Huang et al. used the same dose of ATG as we did [16, 17]. However, in contrast to our findings, they observed a lower cGVHD incidence in patients treated with ATG-F. This discrepancy may be caused by the inadequate number of patients included and the absence of multivariate analysis in their study.

Although studies have shown no difference in T-cell reconstitution and similar GVHD incidence between patients who receiving ATG-F 60 mg/kg and those who receive 45 mg/kg [22], more studies support that lowering the dose of ATG-F improves survival. A retrospective study by Ayuk et al. compared ATG-F 30 mg/kg and 60 mg/kg in cases of unrelated matched transplantation. The results showed that ATG dose had no effect on the rate of GVHD or relapse, but the lower dose was associated with a decreased rate of fatal infection and TRM and increased DFS [26]. ATG-F 35 mg/kg and 60 mg/kg for elderly patients receiving unrelated donor transplantation was compared with no ATG by Binkert et al. TRM and survival in the lower dose group were superior to those in the no ATG group, while the higher dose showed no advantage [27]. A multicenter phase 3 randomized clinical trial by Locatelli et al. showed that there was no difference in the rate of grade II-IV acute GVHD, NRM or the relapse rate between children who received unrelated donor transplantation with ATG-F 15 mg/kg and those who received ATG-F 30 mg/kg. However, the lower dose was related to an increased 5-year OS and EFS [28]. Therefore, reducing the dose of ATG-F may improve survival without increasing the risk of GVHD. In this article, despite the higher risk of extensive cGVHD in patients receiving ATG-F 20 mg/kg, their aGVHD incidence, overall cGVHD incidence and survival were similar to those of patients receiving ATG-G 10 mg/kg.

Analysis of posttransplant lymphocyte subsets clearly showed that higher doses of ATG-G, but not ATG-F, led to delayed immune reconstitution [22, 29, 30]. Therefore, theoretically, higher doses of ATG-G may increase the risk of infection and relapse, but studies have shown an inconsistent effect of higher doses of ATG-G on survival. A randomized controlled clinical study by Wang et al. showed that with a lower incidence of grade III-IV aGVHD, the ATG-G 10 mg/kg group had a similar 1-year DFS to the ATG-G 6 mg/kg group in haploidentical transplantation [31]. The long-term follow-up results of another prospective randomized clinical study showed that although ATG-G 10 mg/kg increased the risk of infection in haploidentical transplantation compared to ATG-G 6 mg/kg, the incidence of cGVHD was decreased, and GRFS was improved [32]. A retrospective study by Devillier et al. found that in reduced-intensity conditioning matched sibling donor transplantation, doses of ATG-G higher than 6 mg/kg led to an adverse impact on survival compared with doses less than 6 mg/kg because of an increased rate of relapse [33]. A randomized controlled clinical study found that 7.5 mg/kg ATG-G did not decrease the incidence of grade III-IV aGVHD. Furthermore, 15 mg/kg ATG-G reduced the incidences of grade III-IV aGVHD and extensive cGVHD but did not improve survival due to an increased incidence of fatal infection compared to no ATG [34]. The contradictory findings of the above study related to higher doses of ATG-G may have been caused by differences in the optimal dose of ATG determined for different donor sources and conditioning intensities. The similar relapse rate and NRM and decreased risk of extensive cGVHD in the ATG-G 10 mg/kg group compared with those in the ATG-F 20 mg/kg group in this study indicate that ATG-G at a dose of 10 mg/kg may be appropriate for unrelated hematopoietic stem cell transplantation.

This study has limitations as a retrospective study. First, it included patients with several diseases because no single disease group had enough patients for analysis, and the disease distribution was different between the ATG-F group and the ATG-G group, which may have influenced the analysis results since the risk stratification for different diseases was heterogeneous. Second, the NIH chronic cGVHD scoring system was not used in this study to distinguish the severity of cGVHD since the cGVHD score for some patients was not available. Finally, although the case number in this study is the largest of studies comparing ATG-F and ATG-G, it is still relatively small.

In conclusion, the results of this study suggest that ATG-G at a dose of 10 mg/kg is more effective in reducing extensive cGVHD than ATG-F at a dose of 20 mg/kg in unrelated hematopoietic stem cell transplantation but increases the risk of cytomegaloviremia. Selection of the ATG preparation according to the incidence of extensive cGVHD of each center and adjustment of the posttransplant management strategy according to the ATG preparation are recommended.
